# Adiponectin up‐regulates the decrease of myocardial autophagic flux induced by β_1_‐adrenergic receptor autoantibody partly dependent on AMPK

**DOI:** 10.1111/jcmm.16807

**Published:** 2021-07-29

**Authors:** Cong Sun, Jiebei Lu, Yaolin Long, Shuai Guo, Weiwei Jia, Na Ning, Haihu Hao, Xiaohui Wang, Yunfei Bian, Huirong Liu, Li Wang

**Affiliations:** ^1^ Department of Pathology Shanxi Medical University Taiyuan China; ^2^ Key Laboratory of Cellular Physiology (Shanxi Medical University) Ministry of Education Taiyuan China; ^3^ Department of Pathology Linfen Central Hospital Linfen China; ^4^ Department of Orthopedics Shanxi Bethune Hospital & Shanxi Academy of Medical Sciences Taiyuan China; ^5^ Department of Cardiology The Second Hospital of Shanxi Medical University Taiyuan China; ^6^ Department of Physiology and Pathophysiology School of Basic Medical Sciences Capital Medical University Beijing China

**Keywords:** Adiponectin, AMPK, Autophagic flux, Cardiac dysfunction, β_1_‐AA

## Abstract

Cardiomyocytes autophagy is essential for maintaining cardiac function. Our previous studies have found that β_1_‐adrenergic receptor autoantibody (β_1_‐AA) induced the decreased myocardial autophagic flux, which resulted in cardiomyocyte death and cardiac dysfunction. And other studies demonstrated that β_1_‐AA induced the decrease of AMPK phosphorylation, the key hub of autophagy pathway, while adiponectin up‐regulated autophagic flux mediated by AMPK. However, it is not clear whether adiponectin improves the inhibition of myocardial autophagic flux induced by β_1_‐AA by up‐regulating the level of AMPK phosphorylation. In this study, it has been confirmed that β_1_‐AA induced the decrease of AMPK phosphorylation level in both vivo and vitro. Moreover, pretreatment of cardiomyocytes with AMPK inhibitor Compound C could further reduce the autophagic flux induced by β_1_‐AA. Adiponectin deficiency could aggravate the decrease of myocardial AMPK phosphorylation level, autophagic flux and cardiac function induced by β_1_‐AA. Further, exogenous adiponectin could reverse the decline of AMPK phosphorylation level and autophagic flux induced by β_1_‐AA and even reduce cardiomyocyte death. While pretreated with the Compound C, the adiponectin treatment did not improve the decreased autophagosome formation, but still improved the decreased autophagosome clearance induced by β_1_‐AA in cardiomyocytes. This study is the first time to confirm that β_1_‐AA could inhibit myocardial autophagic flux by down‐regulating AMPK phosphorylation level. Adiponectin could improve the inhibition of myocardial autophagic flux induced by β_1_‐AA partly dependent on AMPK, so as to provide an experimental basis for the treatment of patients with β_1_‐AA‐positive cardiac dysfunction.

## 1 INTRODUCTION

With the development of the society, the morbidity of cardiac dysfunction is rising year by year, which has become a serious public health concern.^1^ Immune disorder is one of the important causes of cardiac dysfunction.^2^ In 1987, the researcher first discovered β_1_‐adrenergic receptor autoantibody (β_1_‐AA) as a product of immune disorder in serum of patients with dilated cardiomyopathy.^3^ Subsequently, other researchers found that about 40%‐60% of patients with cardiac dysfunction were detected with β_1_‐AA in serum.^4^ It can bind with β_1_‐adrenoceptor (β_1_‐AR) on the surface of cardiomyocyte membrane to cause persistent injury of cardiomyocytes, induce cardiomyocyte death and eventually lead to cardiac dysfunction.^4^ Our team originally found that β_1_‐AA could induce the decrease of myocardial autophagic flux, which contributed to cardiac dysfunction.^5^ Autophagy is a conserved intracellular degradation system, which maintains myocardial function and improves cardiac dysfunction by clearing intracellular degenerative and ageing proteins or organelles.^6^, ^7^ However, it is not clear how β_1_‐AA induces the decreased myocardial autophagic flux.

AMP‐dependent protein kinase (AMP)‐activated protein kinase (AMPK) is an important signal molecule in the upstream of autophagy,^8^ which plays a positive role in regulating autophagic flux.^9^ AMPK phosphorylation activated autophagy via Unc‐51 like autophagy activating kinase 1 (Ulk1)‐Beclin1 signalling pathway and through inhibiting mammalian target of rapamycin (mTOR) activity.^10^ In primary neonatal rat cardiomyocytes, β_1_‐AA induced a decrease in AMPK phosphorylation.^11^ However, whether the decrease of AMPK phosphorylation level is involved in the decline of myocardial autophagic flux induced by β_1_‐AA, and whether the increase of AMPK phosphorylation level could improve the decreased myocardial autophagic flux induced by β_1_‐AA remains to be explored.

As well known, as a key protein of energy metabolism, adiponectin is closely related to the energy metabolism hub AMPK.^12^ It has been reported that adiponectin combined to adiponectin receptor on the surface of breast cancer cells, which led to the decrease of intracellular ATP content, activation of LKB1, then induced AMPK phosphorylation, activation of downstream Ulk1, finally initiation of autophagy.^12^, ^13^ According to the published papers, patients with cardiac dysfunction have hypoadiponectinaemia and adiponectin knockout mice (APN‐KO mice) have serious cardiac dysfunction. Thus, we speculate that adiponectin deficiency leads to cardiac dysfunction. Moreover, adiponectin is an endogenous product of human, which has a wide range of myocardial protection.^14^, ^15^, ^16^ Therefore, we speculate that adiponectin may promote myocardial autophagic flux by up‐regulating the decrease of AMPK phosphorylation level induced by β_1_‐AA, thus improving cardiac dysfunction.

This study has confirmed for the first time that the decrease of AMPK phosphorylation contributed to β_1_‐AA‐induced decrease of myocardial autophagic flux both in vivo and vitro. Then, APN‐KO mice were used to confirm the role of adiponectin deficiency in β_1_‐AA‐induced decline of autophagic flux, cardiac dysfunction and myocardial fibrosis. Pretreatment of adiponectin verified that adiponectin could improve β_1_‐AA‐induced decrease of cardiomyocyte autophagic flux and cardiomyocyte death in vitro. Furthermore, Compound C was used to inhibit the AMPK to clarify the specific mechanism of adiponectin regulating autophagic flux. Our observations may open new insights into the treatment of β_1_‐AA‐positive cardiac dysfunction patients.

## 2 MATERIALS AND METHODS

### 2.1 Experimental animals

Six‐ to eight‐week‐old male APN‐KO mice (weight 18–20 g) were obtained from Professor Bian Yunfei, Second Hospital of Shanxi Medical University. Six‐ to eight‐week‐old male C57BL/6 mice (weight 18‐20 g) were obtained from Animal Center of Shanxi Medical University. The mice with free diet were placed in a suitable temperature and humidity environment throughout the experiment. If the myocardial tissue of mice was taken, 10 % chloral hydrate was used for anaesthesia. All procedures related to animals in this study were approved by the Ethics Committee of Shanxi Medical University and followed the People's Republic of China's Guidelines for the Care and Use of Laboratory Animals.

### 2.2 Agarose gel electrophoresis

The genotypes of APN‐KO mice were identified by agarose gel electrophoresis. The mouse tail tissue about 5 × 10^−3^ m was put into the EP tube; then, the tail lysate was added and put into the 55 ℃ constant temperature water bath for the night. The DNA of mice tail tissue was extracted and amplified, and the primer sequences were as follows: P1: GGCTCTCTGGGAGAGGCGAG, P2: CCATCACGGCCTGGTGTGCC, P3: TTCGCCATTCAGGCTGCGCA. The samples were agarose gel electrophoresis, after electrophoresis, the glue was placed in the automatic exposure instrument.^17^

### 2.3 Establishment of β_1_‐AA actively immunized mouse model

**FIGURE 1:**
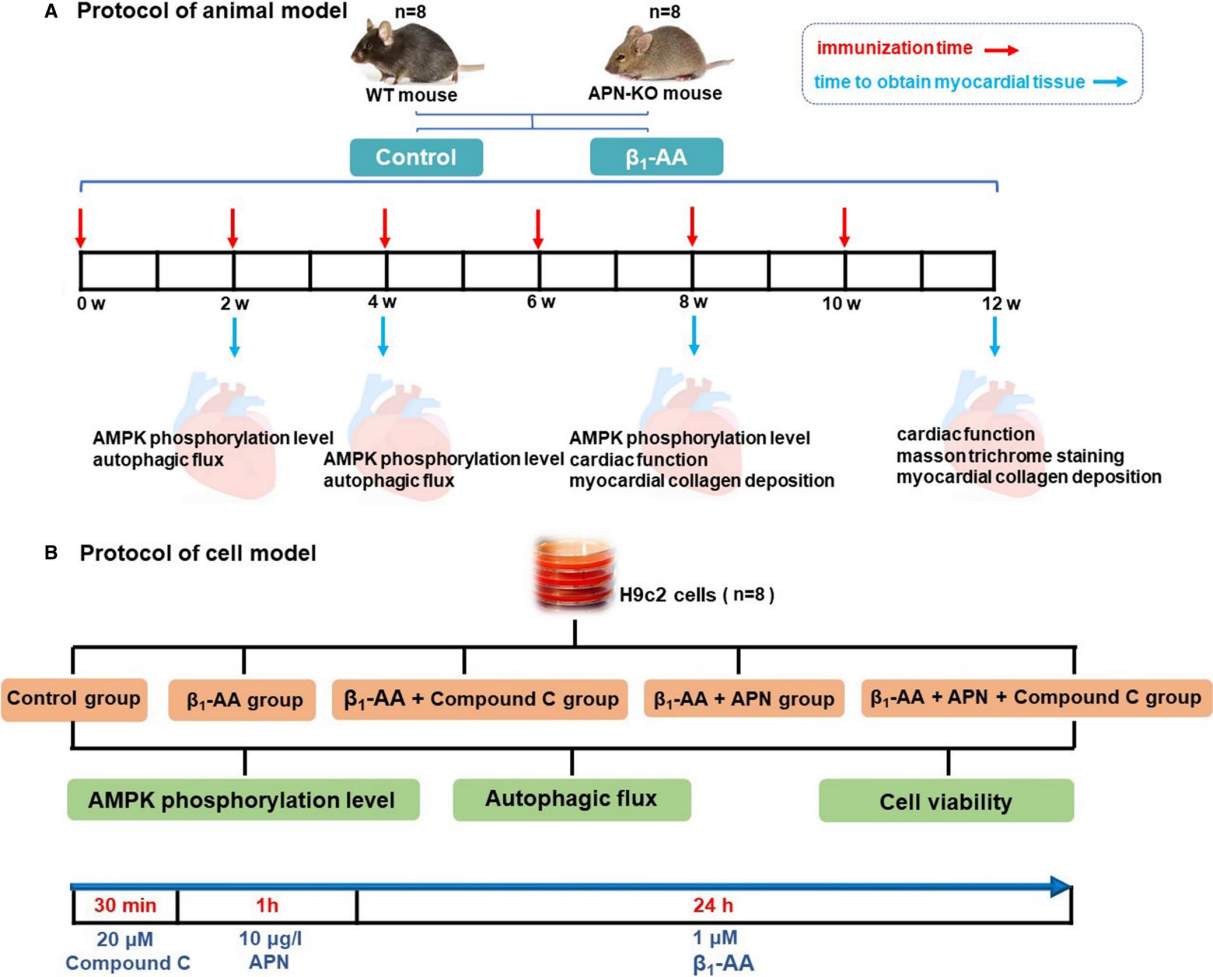
The protocols of vivo model and vitro model. A, The protocols of animal model. B, The protocols of cell model

Six‐ to eight‐week‐old male APN‐KO mice and WT (C57BL/6) mice were randomly divided into actively immunized group and solvent control group, with 8 mice in each group. The peptide of β_1_‐AR‐ECII (GLS, Shanghai, Chinese) was dissolved and diluted with Na_2_CO_3_ solution (100 mM, pH 11.0) and then mixed with Freund's complete adjuvant (Sigma‐Aldrich). After 1:1 emulsification and mixing, the mice were immunized with multiple injection subcutaneously into the back (0.4 μg/g) during the first immunization. Subsequently, diluted peptide of β_1_‐AR‐ECII emulsified with incomplete Freund's adjuvant (Sigma‐Aldrich), and single subcutaneous injection was used to strengthen immunization once every 2 weeks for 12 weeks. In the solvent control group, the same amount of Na_2_CO_3_ solution was used to replace the antigen solution. Compound C group is pretreated with Compound C (0.4 μg/g, IV) (s7840, Selleck, USA) for 10 min before immunization (Figure 1A, Figure S1).^18^

### 2.4 SA‐ELISA

SA‐ELISA was used to detect the level of β_1_‐AA in the serum of actively immunized mice described previously.^5^ The 96‐well plates were used for antigen coating, and blank control group, solvent control group, positive control and actively immunized group were set up. β_1_‐AR‐ECII was dissolved in Na_2_CO_3_ (100 mM, PH = 11.0) to prepare a solution with the final concentration of 10 μg/mL, and 50 μL was added into each well. Except for the blank control group, the other groups were coated with antigen at 4℃ overnight. The wells were saturated with PMT (1% (w/v) bovine serum albumin, 0.1% (v/v) Tween 20 in PBS, PH 7.4). The solvent control group, positive control, blank control and serum to be tested were diluted and added to 96‐well plates. Then, the 96‐well plates were added with biotinylated antibody (Zhongshan Golden Bridge Biotechnology, 1:2000) and horseradish peroxidase streptavidin (Zhongshan Golden Bridge Biotechnology, 1:2000) in 96‐well plates. The above steps were, respectively, incubated at 37℃ for 1 h. The substrate (2.5 mM H_2_O_2_, 2 mM 2, 2'‐azinodi (ethylbenzthiazoline) sulfuric salt (ABTS, Bio Basic Inc., AD0002, Markham, ON, Canada) was added for 30 min. Finally, the 96‐well plates were put into the microplate reader, and the OD value of each group was measured at 405 nm.^18^

### 2.5 Affinity chromatography

First, a β_1_‐AA‐positive animal model was established by actively immunized rats with β_1_‐AR‐ECII, as described in the previous studies.^19^ Then, animal serum from actively immunized rats and the control group was collected and extracted using MAbTrap Kit (GE Healthcare, Uppsala, Sweden) for affinity and purification of IgG.

### 2.6 Cell culture

H9c2 cells were purchased from Chinese Academy of Sciences Cell Bank (Shanghai, China). H9c2 cells were cultured in a complete medium containing 10 % foetal bovine serum (Sijiqing, Shanghai, China) and 100 U/mL penicillin and 100 μg/mL streptomycin (Solarbio, P1400‐100, Beijing, China) and were incubated at 37 ℃ in a humidified atmosphere of 5 % CO_2_. The cells were subcultured every other day. Cell grouping and treatment: control group (treated with 1 μM negative IgG for 24 h), β_1_‐AA group (treated with 1 μM β_1_‐AA for 24 h), β_1_‐AA +adiponectin group (450–27, Pepro tech, USA) (pretreated with 10 μg/L adiponectin for 1 h and then treated with 1 μM β_1_‐AA for 24 h), β_1_‐AA +adiponectin + Compound C (s7840, Selleck, USA) (pretreated with 20 μM Compound C for 30 min, then 10 μg/L adiponectin was added for 1 h before adding 1 μM β_1_‐AA for 24 h), β_1_‐AA +Compound C group (pretreated with 20 μM Compound C for 30 min and then added 1 μM β_1_‐AA for 24 h), Compound C group (pretreated with 20 μM Compound C for 30 min). The sample size is 8 for each experimental group (Figure 1B).^18^

### 2.7 Western blotting

Western blotting was used to detect the protein expression levels of LC3B (ab48394, Abcam, 1:1000), p62 (ab56416, Abcam, 1:1000), AMPK (#5831, CST, 1:1000), p‐AMPK (#2535, CST, 1:1000), collagen I (ab255809, Abcam, 1:1000) and collagen III (ab7778, Abcam, 1:1000) in each group. Samples of each group were collected and added with RIPA lysis buffer (p0013b, Beyond Biotechnology, China) for protein extraction. BCA Kit (23225, Thermo Scientific, Rockford, IL, USA) was used to determine the protein concentration and then boiled to denaturation. The protein was analysed by SDS‐PAGE assay (the sample volume was 40 μg) and transferred to the PVDF membrane (IPVH00010, Millipore, Billerica, MA, USA) and then blocking for 2 h in 5% non‐fat dry milk which was dissolved with 1 × Tris‐buffered saline and incubated with the corresponding antibodies at 4℃ overnight. TBST was used to wash the membranes; then, the membranes were incubated with the corresponding secondary antibodies (zb‐2305, zb‐2301, Zhongshan Golden Bridge Biotechnology, 1:1000) at room temperature. After washing the membrane with TBST, the membranes were placed in the automatic exposure instrument. After adding Super ECL Plus (Applygen Technologies), the blots were exposed and the grey value was analysed by ImageJ. GAPDH was used as internal references to calculate the relative expression of different proteins.^18^

### 2.8 Real‐time PCR

We detected the mRNA levels of autophagy‐related genes Beclin1 and LC3B by real‐time PCR. Firstly, total RNA was extracted by Trizol method; then, the total RNA was reverse‐transcribed to cDNA. We used SYBR Green Kit (Takara, Japan) to amplify. The specific sequence of primers was as follows: Beclin1 (GenBank ID NM_001034117.1), upstream primer: 5′‐GAAACTGGACACGAGCTTCAAGA‐3′, downstream primer: 5′‐ACCATCCTGGCGAGTTTCAATA‐3′. LC3B (GenBank ID NM_022867.2), upstream primer: 5′‐AGCTCTGAAGGCAACAGCAACA‐3′, downstream primer: 5′‐GCTCCATGCAGGTAGCAGGAA‐3′. GAPDH (GenBank ID NM_017008.3), upstream primer: 5′‐GGCACAGTCAAGGCTGAGAATG‐3′, downstream primer: 5′‐ATGGTGGTGAAGACGCCAGTA −3′. GAPDH was selected as the internal control. Data were quantified by the relative quantitative 2^−ΔΔCt^ method.^18^

### 2.9 CCK‐8

Cell viability was measured with a cell counting kit‐8 (CCK‐8). The H9c2 cells were seeded on 96‐well plates (2 × 10^9^/L). After H9c2 cells adhered to the wall, the H9c2 cells were pretreated with 5 μg/L, 10 μg/L and 30 μg/L adiponectin for 1 h before adding 1 μM β_1_‐AA for 24 h. Then, 10 μL CCK‐8 reagent (CK04, Dojindo Molecular Technologies, Kumamoto, Japan) was added into each well. When the colour of the solution changes to brown yellow, the absorbance values of each group at 450 nm were detected by microplate reader. According to the absorbance value of each group, the cell survival rate of each experimental group compared with the control group was calculated as follows: viability %= [(AS−AB)/(AC−AB)] ×100%, where AS is the absorbance of the samples with β_1_‐AA, AC is the absorbance of the DMEM media, and AB is the absorbance of the control.^5^

### 2.10 Small animal ultrasound

The changes of cardiac function in mice were detected by small animal ultrasound. A small animal anaesthesia machine was used for gaseous anaesthesia, then put the mice into the anaesthesia box and wait for the mice to faint. Fix the anaesthetized mice on the mouse plate with adhesive tape, and maintain anaesthesia by inhalation, remove the hair in the precordial area of the mice with depilatory creams and gently wipe the residual hair with three distilled water to avoid causing artefacts. Fix the mice on the ultrasound platform and smear the coupling agent on the precordial area of the mice. The left ventricular ejection fraction (EF%), fractional shortening (FS%), LV internal diameter at end systole (LVID (s)) and LV internal diameter at end diastole (LVID (d)) were measured by M‐mode ultrasound in the short‐axis section of the heart. After the detection, the precordial coupling agent was wiped off and put back into the cage.^20^

### 2.11 Masson trichrome staining

Masson trichrome staining was used to observe the degree of myocardial fibrosis in mice. At the 12 weeks of immunization, mice were killed. The remaining blood in the heart was pumped out in PBS and fixed in 4% paraformaldehyde. The fixed tissue was washed with water and embedded in paraffin and sectioned (4 μm). After dewaxing, the tissue was stained with Masson trichrome stain kit (Solarbio, Beijing, China) and sealed with neutral gum. All tissue slides were analysed by optical microscopy (Olympus, BX45, Olympus Corporation, Tokyo, Japan) by the double‐blind fashion.^20^

### 2.12 Statistical analysis

Data are expressed as means ±SD. Statistical analysis was performed with SPSS software (version 16.0, SPSS Inc., Chicago, IL, USA). Two independent‐sample tests were used to compare the means of two independent samples, and one‐way ANOVA was applied after a Bonferroni post hoc test for more than two samples. *P* < 0.05 was considered statistically significant.

## 3 RESULTS

### 3.1 β_1_‐AA inhibited autophagic flux in myocardial tissues by reducing AMPK phosphorylation

**FIGURE 2:**
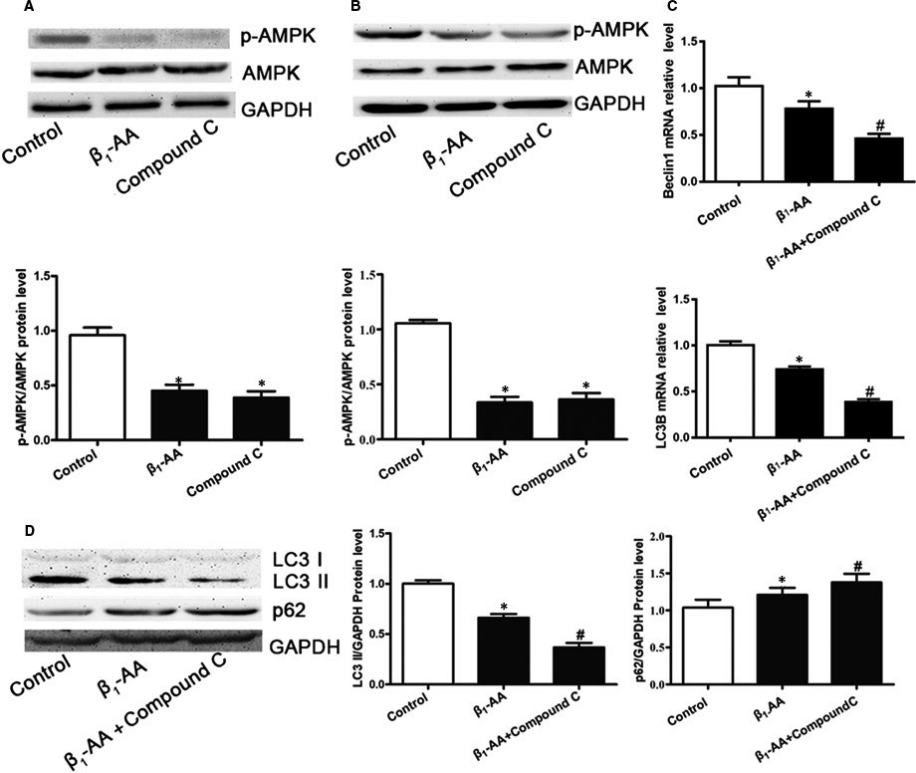
Expression of AMPK phosphorylation was decreased and autophagic flux was further decreased interfered with Compound C after β_1_‐AA stimulation in myocardial tissues. A, The AMPK phosphorylation of myocardial tissues was analysed by Western blotting at 8 weeks after active immunization. B, The AMPK phosphorylation of H9c2 cells was analysed by Western blotting after β_1_‐AA stimulation at 24 h. C, The LC3B and Beclin1 mRNA expression was analysed by real‐time PCR in H9c2 cells pretreated with Compound C after β_1_‐AA stimulation. D, The LC3 II and p62 protein expression was analysed by Western blotting in H9c2 cells pretreated with Compound C after β_1_‐AA stimulation. The values were normalized to GAPDH. *P < 0.05, compared with Control group. ^#^ *P* < 0.05, compared with β_1_‐AA group. Values are means±SD (n = 8)

Firstly, the effect of β_1_‐AA on myocardial AMPK phosphorylation was investigated. The results showed that after active immunization with β_1_‐AR‐ECII for 8 weeks, the AMPK phosphorylation level (p‐AMPK/AMPK) of myocardial tissues was considerably decreased (Figure 2A). We also found that the AMPK phosphorylation level (p‐AMPK/AMPK) of H9c2 cells was also significantly decreased after stimulation of β_1_‐AA for 24 hours (Figure 2B). The results showed that compared with control group, the phosphorylation level of AMPK in Compound C group was significantly decreased both in vitro and in vivo (Figure 2A,B).

In order to clarify the role of decreased AMPK phosphorylation in the decline of autophagic flux induced by β_1_‐AA, H9c2 cells were pretreated with Compound C, an AMPK inhibitor, and then treated with β_1_‐AA for 24 hours. The results showed that the inhibition of AMPK by Compound C further aggravated the decline of Beclin1 and LC3B mRNA levels (Figure 2C) and LC3 II protein levels induced by β_1_‐AA (Figure 2D), and aggravated the accumulation of p62 protein (Figure 2D).

### 3.2 Adiponectin deficiency aggravated the decrease of AMPK phosphorylation induced by β_1_‐AA in myocardial tissues

**FIGURE 3:**
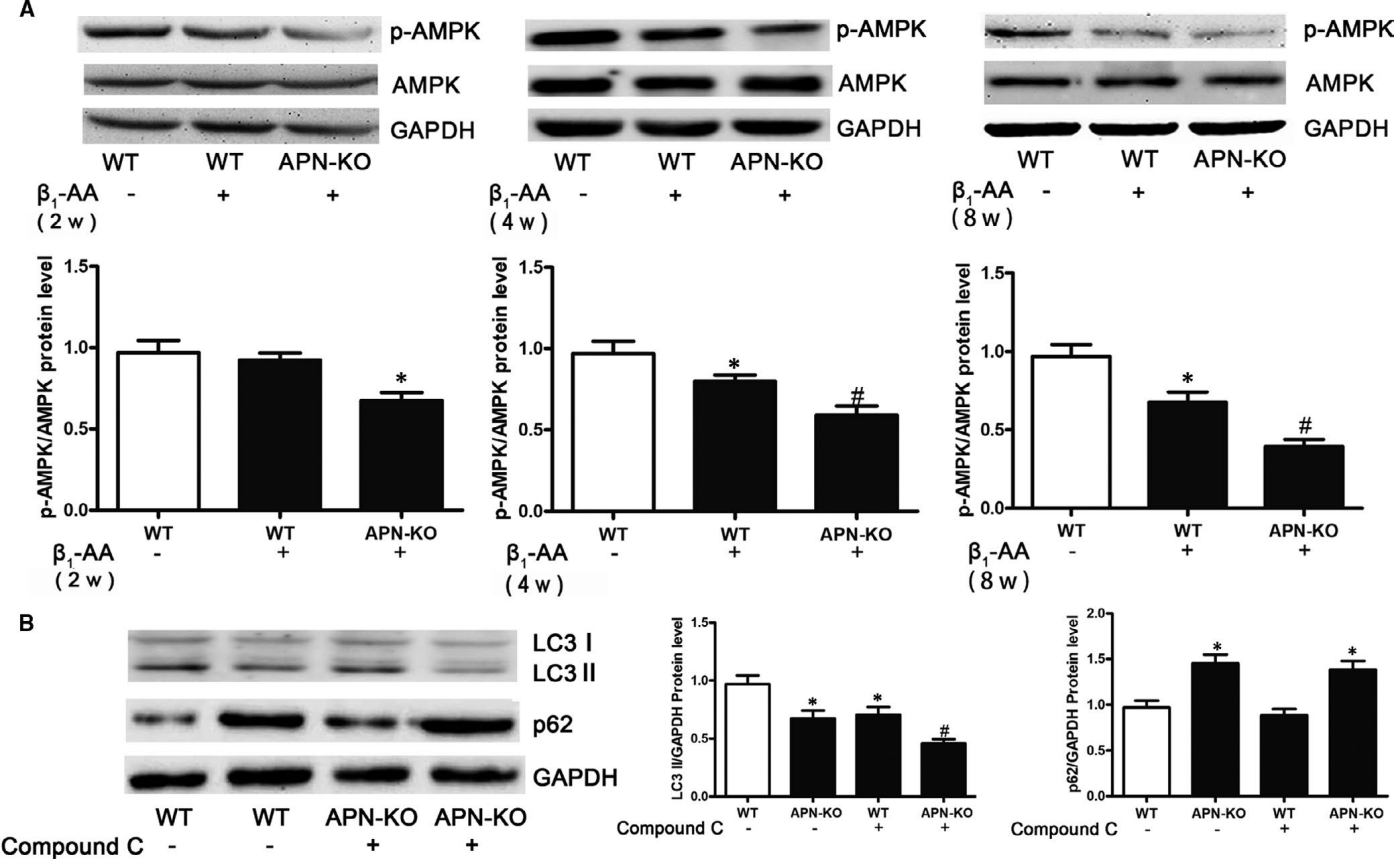
The expression of AMPK phosphorylation was further decreased in APN‐KO mice after β_1_‐AA stimulation in myocardial tissues. A, Expression of AMPK phosphorylation of myocardial tissues was analysed by Western blotting in WT mice and APN‐KO mice at 2 weeks, 4 weeks and 8 weeks after active immunization. B, The LC3 II and p62 protein expression was analysed by Western blotting in WT mice and APN‐KO mice pretreated with Compound C after active immunization. The values were normalized to GAPDH. *P < 0.05, compared with Control group. ^#^ *P* < 0.05, compared with β_1_‐AA group. Values are means±SD (n = 8)

To investigate the effect of adiponectin on β_1_‐AA‐induced decrease of myocardial AMPK phosphorylation, APN‐KO mice was used to confirm the role of adiponectin deficiency on β_1_‐AA‐induced decrease of myocardial AMPK phosphorylation from reverse. First of all, the genotypes of APN‐KO mice were identified by agarose gel electrophoresis to ensure the success of adiponectin gene knockout (Figure S2). After that, APN‐KO mice and WT mice were immunized with β_1_‐AR‐ECII for 2 weeks, 4 weeks and 8 weeks. Western blotting was used to detect the phosphorylation of AMPK in the myocardial tissues. The results showed that compared with the WT group, the myocardial AMPK phosphorylation level of WT actively immunized mice had no statistical difference at 2 weeks (Figure 3A); the myocardial AMPK phosphorylation level of APN‐KO actively immunized mice was decreased (Figure 3A). Compared with the WT group, the myocardial AMPK phosphorylation level of began to decrease at 4 weeks (Figure 3A), the myocardial AMPK phosphorylation level of APN‐KO actively immunized mice was further decreased (Figure 3A). At 8 weeks, compared with the WT group, the myocardial AMPK phosphorylation level of WT‐immunized mice was decreased (Figure 3A), and the myocardial AMPK phosphorylation level of APN‐KO immunized mice was further decreased (Figure 3A).

In order to better assess the role of AMPK in adiponectin effect, we pretreated WT mice and APN‐KO mice with AMPK inhibitor Compound C. The results showed that compared with WT group, Compound C pretreatment (Compound C + WT group) could significantly reduce the level of LC3 II protein in mouse myocardial tissue (Figure 3B). Compared with Compound C pretreatment group, LC3 II protein level was further decreased in Compound C + APN‐KO group (Figure 3B). Compared with the WT group, p62 protein level in APN‐KO group was significantly increased (Figure 3B), and Compound C had no effect on p62 protein expression in WT group and APN‐KO group (Figure 3B).

### 3.3 Adiponectin deficiency aggravated the decline of autophagic flux induced by β_1_‐AA in myocardial tissues

**FIGURE 4:**
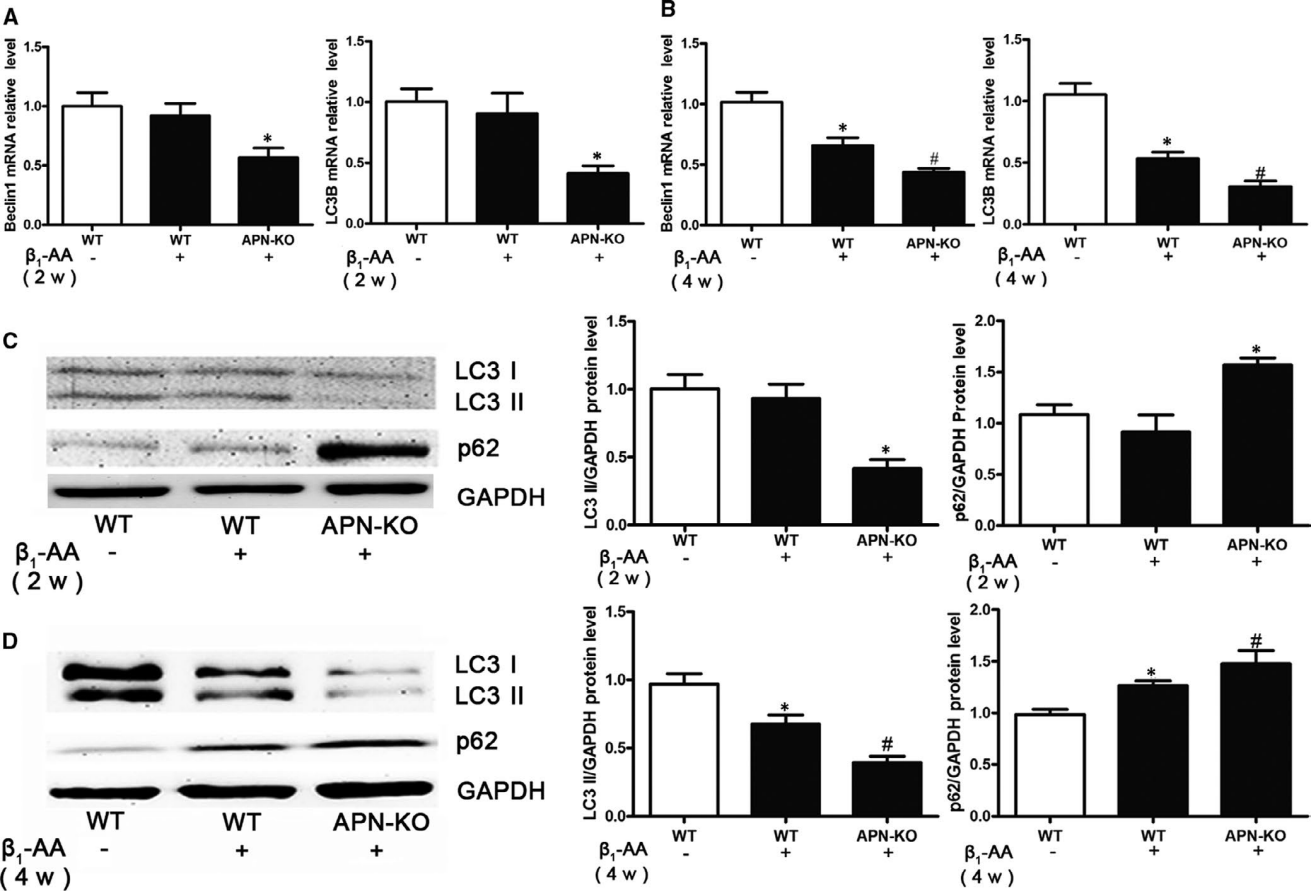
The autophagic flux was further decreased in APN‐KO mice after β_1_‐AA stimulation in myocardial tissues. A, The LC3B and Beclin1 mRNA expression was analysed by real‐time PCR in WT mice and APN‐KO mice at 2 weeks after active immunization. B, The LC3B and Beclin1 mRNA expression was analysed by real‐time PCR in WT mice and APN‐KO mice at 4 weeks after active immunization. C, The LC3 II and P62 protein expression was analysed by Western blotting at 2 weeks after active immunization. D, The LC3 II and p62 protein expression was analysed by Western blotting at 4 weeks after active immunization. The values were normalized to GAPDH. *P < 0.05, compared with Control group. ^#^ *P* < 0.05, compared with β_1_‐AA group. Values are means±SD (n = 8)

In order to clarify the role of adiponectin in the decrease of myocardial autophagic flux induced by β_1_‐AA, real‐time PCR and Western blotting were used to detect the changes of autophagy‐related genes and proteins in the myocardial tissues of APN‐KO mice and WT mice after active immunization for 2 weeks. The results showed that the mRNA levels of Beclin1 and LC3B (Figure 4A) and protein levels of LC3 II in APN‐KO actively immunized mice were significantly decreased (Figure 4C) and the protein levels of p62 were significantly increased (Figure 4C) compared with WT group, but there were no significant differences in autophagy‐related genes and proteins between WT actively immunized mice and WT group (Figure 4A, C).

Then, we continued to immunize APN‐KO mice and WT mice for 4 weeks. The autophagic flux of APN‐KO actively immunized mice was further decreased than that of WT actively immunized mice at 4 weeks (Figure 4B,D).

### 3.4 Adiponectin deficiency aggravated cardiac dysfunction induced by β_1_‐AA

**FIGURE 5:**
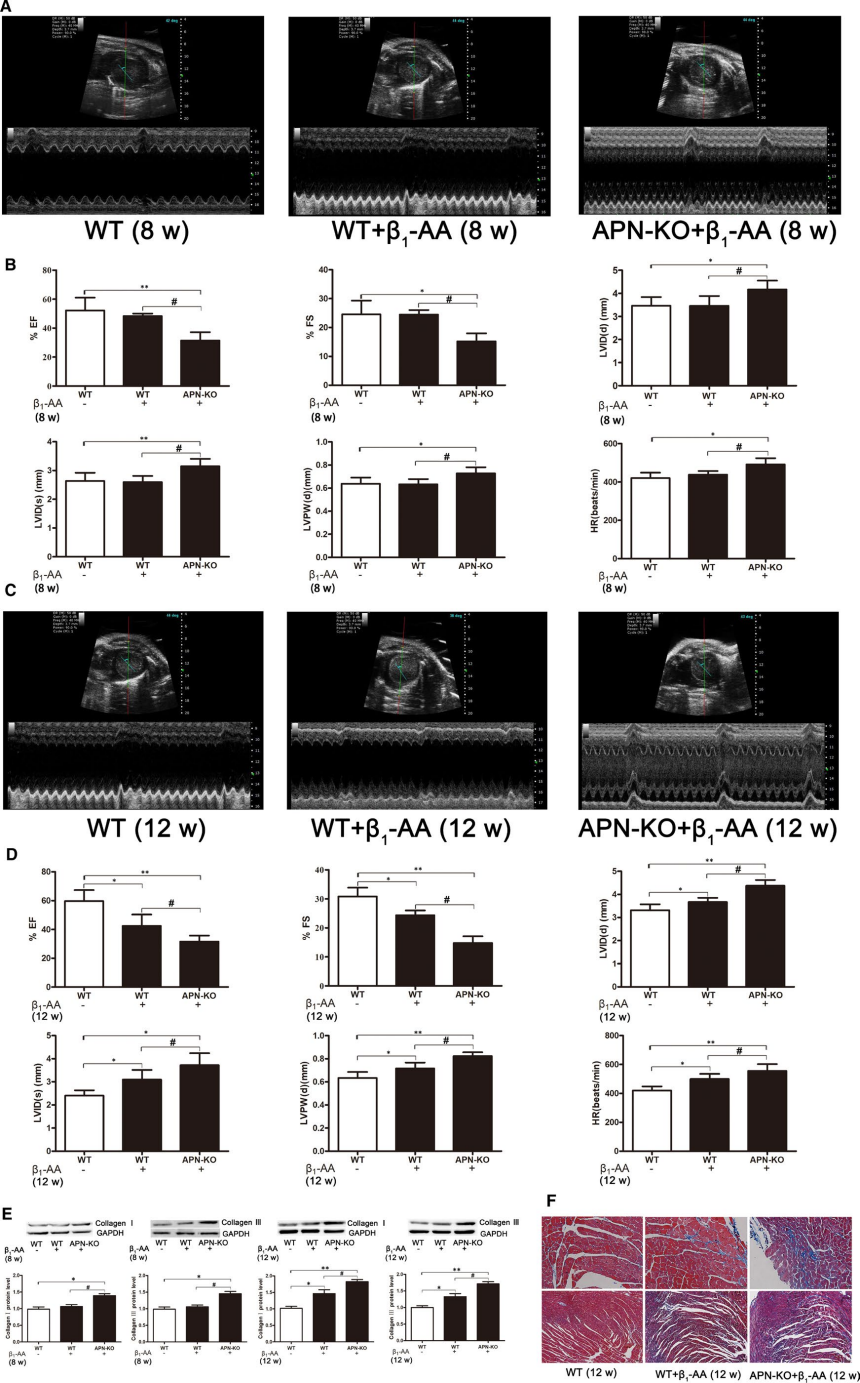
The cardiac dysfunction was aggravated in APN‐KO mice after β_1_‐AA stimulation in myocardial tissues. A, Images are representative of echocardiogram at the 8 weeks after active immunization. B, The left ventricular ejection fraction (EF%), fractional shortening (FS%), LV internal diameter at end systole (LVID (s)) and LV internal diameter at end diastole (LVID (d)) were detected by small animal ultrasound in WT mice and APN‐KO mice at 8 weeks after active immunization. C, Images are representative of echocardiogram at the 12 weeks after active immunization. D, The EF%, FS%, LVID (s) and LVID (d) were detected by small animal ultrasound in WT mice and APN‐KO mice at 12 weeks after active immunization. E, The expression of collagen I and collagen III was observed by Western blotting in WT mice and APN‐KO mice at 8 weeks and 12 weeks after active immunization. F, The myocardial fibrosis was observed by Masson trichrome in WT mice and APN‐KO mice at 12 weeks after active immunization. The values were normalized to Control group. *P < 0.05, **P < 0.01, compared with Control group. ^#^ *P* < 0.05, compared with β_1_‐AA group. Values are means±SD (n = 8)

To investigate the role of adiponectin in the cardiac dysfunction induced by β_1_‐AA, small animal ultrasound was used to detect the changes of cardiac function in WT mice and APN‐KO mice after active immunization for 8 weeks. The results indicated that compared with the WT group, the left ventricular ejection fraction (EF%) and fractional shortening (FS%) were significantly decreased, LV internal diameter at end systole (LVID (s)), LV internal diameter at end diastole (LVID (d)), heart rate and LV end‐diastolic thickness (LVPW (d)) were significantly increased in APN‐KO actively immunized mice (Figure 5A,B), but there was no significant difference between WT actively immunized mice and the WT group (Figure 5A,B).

Then, we continued to immunize APN‐KO mice and WT mice for 12 weeks. The results indicated that compared with WT actively immunized mice, EF% and FS% of APN‐KO actively immunized mice were further decreased, LVID (s), LVID (d), heart rate and LVPW (d) were further increased in APN‐KO actively immunized at the 12 weeks after active immunization (Figure 5C,D). Therefore, the heart function of APN‐KO mice was further deteriorated with the prolongation of immunization time.

After 8 weeks and 12 weeks of active immunization, the myocardial tissue of mice in each group was taken and the degree of myocardial fibrosis was detected by Western blotting and Masson trichrome staining. The Western blotting results indicated that compared with WT group, the expression of collagen I and collagen III was increased in APN‐KO actively immunized mice at 8 weeks (Figure 5E). But there was no significant difference between WT actively immunized mice and the WT group 8 weeks (Figure 5E). At 12 weeks, compared with WT actively immunized mice, the expression of collagen I and collagen III was further increased in APN‐KO actively immunized (Figure 5E). The Masson trichrome staining results showed that the degree of myocardial fibrosis (blue‐stained area) in APN‐KO actively immunized mice was more severe than that of WT actively immunized mice (Figure 5F).

### 3.5 Adiponectin improved β_1_‐AA‐induced H9c2 cell death

**FIGURE 6:**
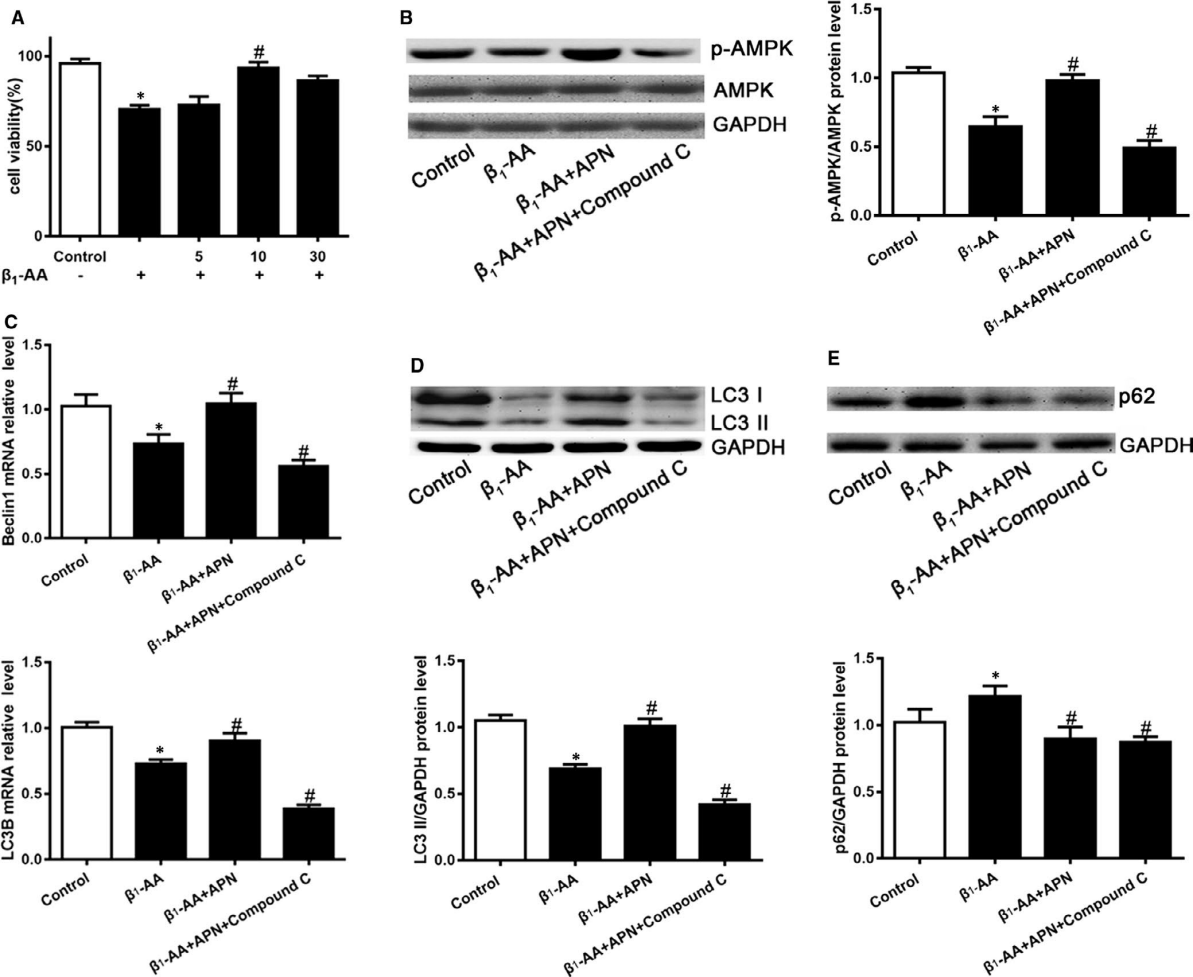
Adiponectin up‐regulated β_1_‐AA‐induced the decreased of H9c2 cell viability and autophagic flux, which is partly dependent on AMPK. A, The cell viability of H9c2 cells was determined by CCK‐8 pretreatment with adiponectin before adding β_1_‐AA. B, The AMPK phosphorylation of H9c2 cells was analysed by Western blotting pretreatment with adiponectin, or adiponectin +Compound C before adding β_1_‐AA. C, The LC3B and Beclin1 mRNA expression of H9c2 cells was analysed by real‐time PCR pretreatment with adiponectin, or adiponectin +Compound C before adding β_1_‐AA. D The LC3 II protein expression of H9c2 cells was analysed by Western blotting pretreatment with adiponectin, or adiponectin +Compound C before adding β_1_‐AA. E, The p62 protein expression of H9c2 cells was analysed by Western blotting pretreatment with adiponectin, or adiponectin +Compound C before adding β_1_‐AA. The values were normalized to GAPDH. *P < 0.05, compared with Control group. ^#^ *P* < 0.05, compared with β_1_‐AA group. Values are means±SD (n = 8)

H9c2 cells were pretreated with different concentrations of adiponectin to explore whether adiponectin could improve the decline in the cell viability induced by β_1_‐AA. The results showed that compared with the control group, the cell viability of H9c2 cells was decreased significantly after β_1_‐AA stimulation, and 10 μg/l adiponectin could significantly reverse the decline of cell viability induced by β_1_‐AA. While 5 μg/l adiponectin could not restore the decreased cell viability. 30 μg/l adiponectin showed a trend of recovery, but there was no significant difference compared with β_1_‐AA group (Figure 6A). The above results suggested that 10 μg/l adiponectin had a significant myocardial protective effect.

### 3.6 Adiponectin up‐regulated the decreased autophagic flux in H9c2 cells after β_1_‐AA stimulation, which was partly dependent on AMPK

In order to verify whether adiponectin could up‐regulate the decrease of autophagic flux induced by β_1_‐AA in H9c2 cells. We pretreated H9c2 cells with adiponectin to detect the changes of autophagic flux. Compared with β_1_‐AA group, adiponectin pretreatment could reverse the decrease of Beclin1 and LC3B mRNA levels (Figure 6C), LC3 II protein levels (Figure 6D) and p62 protein accumulation (Figure 6E) after β_1_‐AA treatment, suggesting that adiponectin could improve the decline of autophagic flux induced by β_1_‐AA.

In order to verify whether adiponectin restored autophagy flux by up‐regulating AMPK phosphorylation, H9c2 cells were pretreated with Compound C, an AMPK inhibitor. The results showed that adiponectin could not improve the decrease of AMPK phosphorylation induced by β_1_‐AA after Compound C pretreatment (β_1_‐AA +APN + Compound C group) (Figure 6B), suggesting that adiponectin could not up‐regulate AMPK phosphorylation when AMPK was inhibited. Meanwhile, adiponectin could not reverse the decrease of Beclin1, LC3B mRNA (Figure 6C) and LC3 II protein (Figure 6D) induced by β_1_‐AA after Compound C pretreatment that means adiponectin up‐regulated the decrease of autophagosome formation dependent on AMPK. However, adiponectin could still improve the accumulation of p62 protein induced by β_1_‐AA after Compound C pretreatment (Figure 6E), suggesting that adiponectin up‐regulated the decrease of autophagosome clearance independent on AMPK. These results suggested that adiponectin up‐regulated decreased autophagic flux in H9c2 cells after β_1_‐AA treatment, which was partly dependent on AMPK.

## 4 DISCUSSION

We have provided the first evidence that β_1_‐AA induced a decrease in myocardial autophagic flux by lowering AMPK phosphorylation. Meantime, we confirmed that adiponectin deficiency aggravated a decrease in myocardial autophagic flux induced by β_1_‐AA. We further proved that adiponectin pretreatment could reverse the decline of myocardial AMPK phosphorylation and autophagy flux after β_1_‐AA treatment, which in turn improved H9c2 cell death. Subsequently, adopting AMPK inhibitor Compound C pretreatment with H9c2 cells, we found that adiponectin up‐regulated decreased autophagic flux induced by β_1_‐AA, which partly depended on the AMPK.

Cardiac dysfunction has become one of the main cause of people's death and lays a great burden on people's economy.^21^ Immune disorders are one of the important causes of heart dysfunction.^2^ Clinical studies have shown that β_1_‐AA, products of immune disorders, exists in the serum of patients with dilation cardiomyopathy and a variety of cardiovascular diseases.^22^, ^23^ As an autoantibody against β_1_‐adrenoceptor, β_1_‐AA binds with β_1_‐AR on the surface of cardiomyocytes and plays an adrenergic receptor agonist like effect of positive chronotropic, positive inotropic and positive transformation conduction, which results in cardiomyocyte death and even cardiac dysfunction.^24^ Our team has originally found that the decrease of myocardial autophagic flux induced by β_1_‐AA is a pivotal cause of cardiomyocyte death and even cardiac dysfunction.^5^ Autophagy is a mechanism to maintain cellular homeostasis. Autophagosome encapsulates cytoplasm, malformed proteins, long‐lived proteins, and organelles and then fuses with lysosomes for degradation.^6^ Additionally, up‐regulation of myocardial autophagic flux with autophagy agonists could improve β_1_‐AA‐induced cardiac dysfunction, suggesting that β_1_‐AA‐induced decline in myocardial autophagic flux is involved in the occurrence and development of cardiac dysfunction.^5^ However, the mechanism by which β_1_‐AA induces the decline of myocardial autophagic flux is yet unclear.

As an important upstream signal molecule of autophagy, AMPK could directly activate autophagy initiation complex Ulk1 and phosphorylate downstream Beclin1 to start autophagy, or inhibit mTOR activity to activate autophagy.^10^ AMPK was originally defined as a protein kinase derived from rat liver. It exists as a trimeric complex consisting of catalytic α‐subunits and regulatory β‐ and γ‐subunits.^25^ When the cellular energy status changes, the upstream kinase liver kinase B1 (LKB1) phosphorylates the threonine residues (Thr‐172) in the kinase domain to activate AMPK.^26^ The study revealed that LPS induced a decrease in AMPK phosphorylation in acute lung injury, which resulted in autophagy inhibition. While the phosphorylation level of AMPK was restored, the decreased autophagy was also improved.^27^ Therefore, the decrease of AMPK phosphorylation level may be an important reason for autophagy inhibition. This study and other studies have confirmed that β_1_‐AA could induce a decrease in the phosphorylation of myocardial AMPK in vivo and in vitro.^11^ We speculated that β_1_‐AA induced a decline in myocardial autophagic flux due to inhibiting the phosphorylation of AMPK. In order to verify this speculation, we used AMPK inhibitor Compound C to pretreat H9c2 cardiomyocytes before adding β_1_‐AA to detect changes in autophagic flux. It is worth noting that the autophagic flux is a dynamic process, including the formation and maturation of autophagosomes, combining with lysosomes to form autophagolysosomes and finally degrading the contents in an acidic environment to maintain the cell's homeostasis.^28^ Beclin1, LC3 II and p62 are commonly autophagy makers.^29^ Beclin1, LC3 II proteins are association with the formation of autophagosomes.^30^, ^31^ Reduction in protein expression of p62, the substrate of autophagy, represents the clearance of autophagosomes.^32^ From the formation of autophagosomes to the clearance of autophagosomes, it is a complete autophagic flux.^28^ The results demonstrated that inhibition of AMPK could further aggravate the decrease of Beclin1 and LC3B mRNA and LC3 II protein level induced by β_1_‐AA and aggravate the accumulation of p62 protein, suggesting that β_1_‐AA reduced myocardial autophagic flux by inhibiting AMPK phosphorylation. If we could effectively up‐regulate the level of AMPK phosphorylation in cardiomyocytes, it is possible to improve the inhibition of autophagic flux induced by β_1_‐AA.

Adiponectin could up‐regulate AMPK phosphorylation and then up‐regulate autophagy. The evidence demonstrates that adiponectin up‐regulates the autophagic flux of skeletal muscle cells by phosphorylating AMPK^33^ and also activates autophagy through the AMPK to reduce the apoptosis of HepG2 cells.^34^ Adiponectin is an adipokine that binds to adiponectin receptors and induces AMPK phosphorylation, followed by activated ULK1 kinase complex which is an important kinase complex for autophagy initiation, finally phosphorylates Beclin1 to initiate autophagy.^12^, ^13^ Therefore, adiponectin up‐regulates autophagic flux through AMPK‐ULK1. Other study demonstrated that adiponectin could induce AMPK phosphorylation in the mouse hypothalamus, and in the hypothalamus of APN‐KO mice, the level of AMPK phosphorylation is significantly decreased,^35^ suggesting that adiponectin deficiency induced the decrease in AMPK phosphorylation. Of course, the level of adiponectin change is a very important point in β_1_‐AR‐ECII active immunization model. We tested the serum adiponectin level, compared with the control group, the serum adiponectin level of β_1_‐AA active immunized mice was significantly increased (Figure S3). It was reported that there was no significant change in the level of adiponectin protein in myocardial tissue of β_1_‐AA active immunized mice at 4 weeks and 8 weeks.^11^ Other study reported that the changes of cardiac adiponectin level and circulating adiponectin level were different in the porcine animal with cardiac dysfunction. The circulating adiponectin level increased, but the cardiac adiponectin level decreased. The circulating adiponectin increase could be a compensatory effect in order to restore metabolic homeostasis. However, the increased circulating adiponectin did not play a role in myocardial protection, and the decrease of cardiac adiponectin is the reason for the decline of heart function.^36^ Subsequently, in order to clarify the correlation between adiponectin and the decrease of AMPK phosphorylation level induced by β_1_‐AA, we actively immunized APN‐KO mice and WT mice with β_1_‐AR‐ECII to detect the changes of AMPK phosphorylation level in the myocardial tissue. The results showed that the phosphorylation level of myocardial AMPK in actively immunized APN‐KO mice was further reduced than that of actively immunized WT mice with the prolongation of immunization time, suggesting that adiponectin deficiency aggravated the decrease in myocardial AMPK phosphorylation induced by β_1_‐AA. In order to better assess the role of AMPK in adiponectin effect, APN‐KO mice and WT mice after actively immunization were pretreated with Compound C. The results suggested that APMK inhibition further decreased the autophagosome formation induced by β_1_‐AA in actively immunized APN‐KO mice. However, APMK inhibition had no effect on the decreased autophagosome clearance induced by β_1_‐AA in actively immunized APN‐KO mice. Subsequently, we further explored the effect of adiponectin deficiency on myocardial autophagy induced by β_1_‐AA.

Studies have shown that APN‐KO mice existed a decreased myocardial autophagic flux, which included inhibition of autophagosome formation and clearance.^37^ And impaired autophagic flux resulted in insulin resistance in skeletal muscle of APN‐KO mice.^38^ As well known, adiponectin is a key factor in regulating metabolism. Adiponectin can increase insulin sensitivity and improve insulin resistance in diabetic patients. The lack of adiponectin leads to PPAR‐α combination decreased, fatty acid and energy consumption decreased, triglyceride content increased, insulin sensitivity in liver and skeletal muscle decreased, and insulin resistance occurred. And myocardial fatty acid β‐oxidation increased, glucose utilization decreased, and myocardial oxygen consumption and load increased, left ventricular enlargement and cardiac dysfunction, resulting in ventricular remodelling.^38^, ^39^ In order to confirm the role of adiponectin deficiency on myocardial autophagic flux induced by β_1_‐AA, we determined the changes in myocardial autophagic flux in actively immunized APN‐KO mice and WT mice. The results demonstrated that actively immunized APN‐KO mice showed decreased myocardial autophagic flux before WT mice, and the myocardial autophagic flux of APN‐KO mice was lower than that of WT mice at 4 weeks, suggesting that adiponectin deficiency significantly aggravated β_1_‐AA induced the decline in myocardial autophagic flux. Additionally, the decrease in myocardial autophagic flux could further cause obesity, metabolic disorders, cardiac hypertrophy and myocardial contractile dysfunction.^16^ Therefore, we are going to prove the effect of adiponectin deficiency on β_1_‐AA‐induced cardiac dysfunction.

Animal experiments showed that APN‐KO mice had severe cardiac dysfunction.^37^ Some studies found that three weeks after transverse aortic constriction (TAC), cardiac remodelling and myocardial systolic dysfunction were significantly greater in APN‐KO mice than WT mice, indicating that adiponectin deficiency led to progressive cardiac remodelling and myocardial systolic dysfunction induced by hypertension.^40^ Moreover, adiponectin deficiency could promote angiotensin II‐induced myocardial fibrosis and then led to cardiac dysfunction.^41^In this study, the changes of cardiac function in APN‐KO mice and WT mice were detected by small animal ultrasound. The indexes included left ventricular ejection fraction (EF%), fractional shortening (FS%), LV internal diameter at end systole (LVID (s)), LV internal diameter at end diastole (LVID (d)), heart rate and LV end‐diastolic thickness (LVPW (d)). Among them, EF% is the measurement of how much blood is being pumped out of the left ventricle of the heart (the main pumping chamber) with each contraction, which is the central measure of left ventricular systolic function.^42^ FS% is used to estimate systolic function. LVID (s), LVID (d) and LVPW (d) are used to assess the changes of cardiac function through the changes of ventricular volume, and the increase of these two indicators represents the decline of cardiac function. In brief, EF%, FS% and LVID (s) represent cardiac systolic function and LVID (d) represents diastolic function.^43^ The increased LVPW (d) represents cardiac hypertrophy.^44^ The results showed that at 8 weeks of active immunization, APN‐KO mice developed cardiac dysfunction earlier than WT mice. And continued active immunization to 12 weeks, APN‐KO mice had further decreased cardiac function compared with WT mice. It is suggested that adiponectin deficiency aggravated the cardiac dysfunction induced by β_1_‐AA. In addition, myocardial fibrosis is also an indicator of cardiac function. Myocardial fibrosis refers to the excessive deposition of collagen in myocardial tissue and the imbalance of the proportion of various types of collagen. There are five types of myocardial collagen; collagen type I and type III are major components of the collagen network. The abnormal increase of collagen fibres could reduce the ventricular compliance and lead to the limitation of ventricular diastolic filling. If the myocardial fibrosis is further aggravated, it will inevitably damage the myocardial contractile function.^45^ We used Western blotting to detect the expression of collagen I and collagen III in mouse myocardial tissue at 8 weeks and 12 weeks. The results indicated that deposition of collagen I and collagen III in APN‐KO actively immunized mice was more serious than that of WT‐immunized mice. After that, the myocardial tissues of mice in each group were collected for 12 weeks. Then, Masson trichrome staining was used to detect the degree of myocardial fibrosis. The results found that the myocardial fibrosis of APN‐KO immunized mice was more serious than that of WT‐immunized mice. These results indicated that adiponectin deficiency aggravated the decline of myocardial autophagic flux induced by β_1_‐AA and then led to cardiac dysfunction and myocardial fibrosis. Therefore, whether adiponectin can be used as a potential therapeutic target to up‐regulate myocardial autophagic flux and improve cardiac dysfunction, and whether AMPK is a possible regulatory mechanism still needs to be further verified.

Then, we pretreated H9c2 cells with adiponectin before adding β_1_‐AA; the results showed that adiponectin indeed up‐regulated the decreased AMPK phosphorylation and inversed the inhibited formation of autophagosomes and the accumulation of p62 protein. These results indicated that adiponectin could improve the decrease in myocardial autophagic flux induced by β_1_‐AA. In order to verify the role of AMPK phosphorylation in the up‐regulation of myocardial autophagic flux by adiponectin, H9c2 cells were pretreated with Compound C as an AMPK inhibitor and found that adiponectin did not improve the decreased mRNA expression of LC3B, Beclin1 and protein expression of LC3 II, indicating that adiponectin improved the inhibited formation of autophagosomes induced by β_1_‐AA dependent on AMPK. To our surprise, adiponectin improved the accumulation of p62 protein, which was unaffected by Compound C, suggesting that adiponectin promoted autophagosomes clearance independent on AMPK. There is evidence that in the intestinal tract of drosophila melanogaster, excessive ROS production could cause p62 protein accumulation, leading to autophagy deficiency.^46^ Other studies have also shown that adiponectin receptor agonist could increase the clearance of autophagosome by reducing the production of ROS in myocardial ischaemia and reperfusion of mice.^33^ Therefore, we speculate that adiponectin may increase the clearance of autophagosome by reducing the production of ROS. Taken together, adiponectin improved the decreased autophagic flux induced by β_1_‐AA partly dependent on AMPK. It has been reported that adiponectin could improve myocardial ischaemia‐reperfusion injury in rats through AMPK pathway.^47^ Furthermore, we also found that adiponectin improved myocardial cell death induced by β_1_‐AA, suggesting adiponectin played a protective role in the myocardial.

## 5 CONCLUSION

**FIGURE 7:**
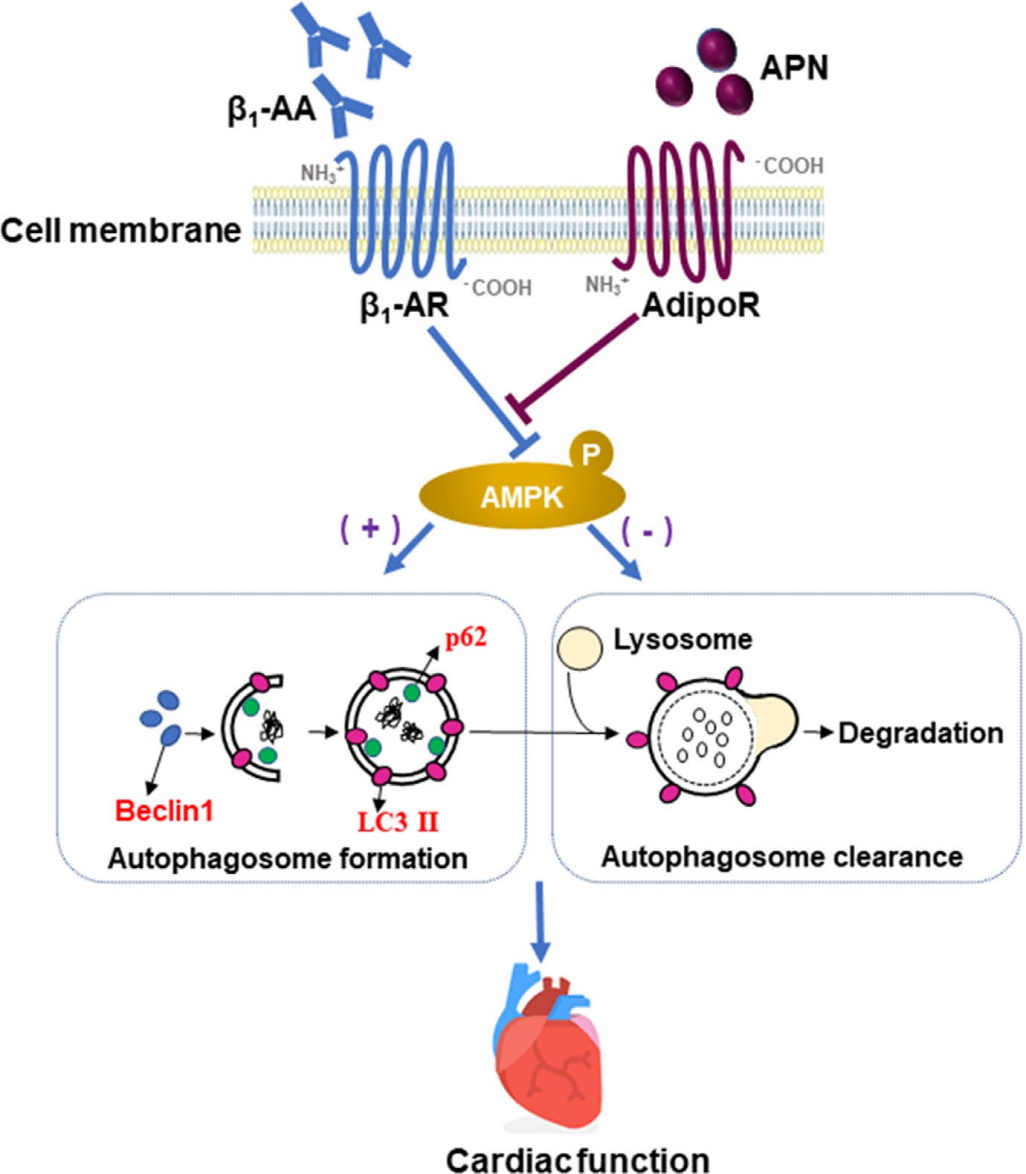
The proposed molecular pathway. β_1_‐AA induced a decrease in myocardial autophagic flux by lowering AMPK phosphorylation. However, adiponectin improved the inhibited formation of autophagosomes induced by β_1_‐AA dependent on AMPK, but adiponectin promoted autophagosomes clearance independent on AMPK

This study is the first to confirm that adiponectin could improve the decrease of myocardial autophagic flux induced by β_1_‐AA, which is partly dependent on AMPK. Firstly, the decrease of AMPK phosphorylation is an important mechanism of the decrease of myocardial autophagic flux induced by β_1_‐AA. Further, it was confirmed that adiponectin deficiency aggravated the decrease of β_1_‐AA‐induced myocardial autophagic flux and further aggravated cardiac dysfunction in vivo. Subsequently, we found that adiponectin pretreatment could indeed improve the decrease of cardiomyocyte autophagic flux induced by β_1_‐AA, which was partly dependent on AMPK. The purpose of this study is to provide an experimental basis for the treatment of β_1_‐AA‐positive patients with cardiac dysfunction from the perspective of improving myocardial autophagic flux (Figure 7).

## CONFLICT OF INTERESTS

The authors declare that they have no competing interest.

## AUTHOR CONTRIBUTION

**Cong Sun:** Formal analysis (equal); Investigation (equal). **Jiebei Lu:** Investigation (equal). **Yaolin Long:** Investigation (equal). **Shuai Guo:** Investigation (equal). **Weiwei Jia:** Investigation (equal). **Na Ning:** Investigation (equal). **Haihu Hao:** Resources (equal). **Xiaohui Wang:** Resources (equal). **Yunfei Bian:** Resources (equal). **Huirong Liu:** Resources (equal). **Li Wang:** Conceptualization (equal); Funding acquisition (equal); Writing‐original draft (equal); Writing‐review & editing (equal).

## ETHICAL APPROVAL

Not applicable.

## CONSENT FOR PUBLICATION

Not applicable.

## Supporting information

Fig S1

Fig S2

Fig S3

## Data Availability

The datasets used and/or analysed during the current study from the corresponding author on reasonable request.
