# Supplementary figures and images for: Adiponectin up‐regulates the decrease of myocardial autophagic flux induced by β1‐adrenergic receptor autoantibody partly dependent on AMPK
Source: J Cell Mol Med. 2021 Jul 29;25(17):8464–78. doi: 10.1111/jcmm.16807 (PMC8419161; doi:10.1111/jcmm.16807)

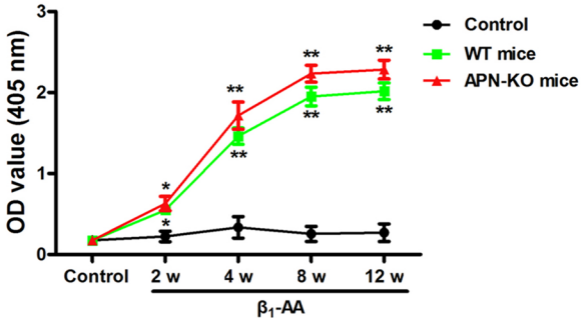

Supplement: Supplementary file 1 — Fig S1 [file JCMM-25-8464-s001.pdf]

**531 bp**

**326 bp**

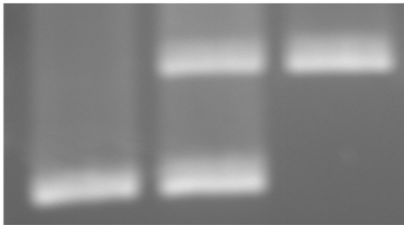

**WT**

**Hete**

**APN-KO**

Supplement: Supplementary file 2 — Fig S2 [file JCMM-25-8464-s002.pdf]

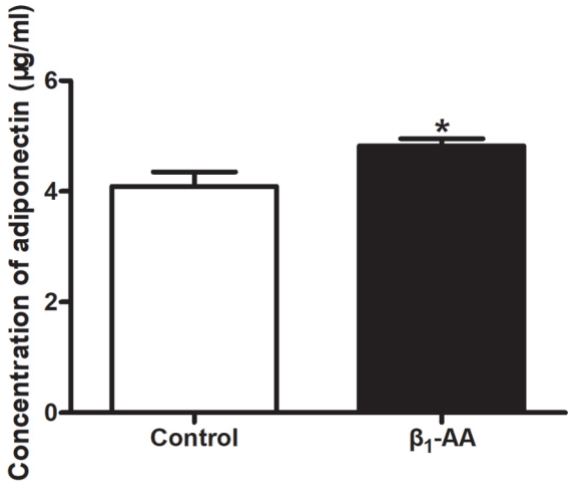

Supplement: Supplementary file 3 — Fig S3 [file JCMM-25-8464-s003.pdf]
